# Evolutionary origins of synchronization for integrating information in neurons

**DOI:** 10.3389/fncel.2024.1525816

**Published:** 2025-01-06

**Authors:** Takashi Shibata, Noriaki Hattori, Hisao Nishijo, Tsutomu Takahashi, Yuko Higuchi, Satoshi Kuroda, Kaoru Takakusaki

**Affiliations:** ^1^Department of Neurosurgery, Toyama University Hospital, Toyama, Japan; ^2^Department of Neurosurgery, Toyama Nishi General Hospital, Toyama, Japan; ^3^Department of Rehabilitation, Toyama University Hospital, Toyama, Japan; ^4^Faculty of Human Sciences, University of East Asia, Yamaguchi, Japan; ^5^Department of Neuropsychiatry, Graduate School of Medicine and Pharmaceutical Sciences, University of Toyama, Toyama, Japan; ^6^Research Center for Idling Brain Science, University of Toyama, Toyama, Japan; ^7^The Research Center for Brain Function and Medical Engineering, Asahikawa Medical University, Asahikawa, Japan

**Keywords:** synchronization, neuron assemblies, binding problem, molecular evolution, information integration, GABAergic inhibitory interneurons, quantum coherence

## Abstract

The evolution of brain-expressed genes is notably slower than that of genes expressed in other tissues, a phenomenon likely due to high-level functional constraints. One such constraint might be the integration of information by neuron assemblies, enhancing environmental adaptability. This study explores the physiological mechanisms of information integration in neurons through three types of synchronization: chemical, electromagnetic, and quantum. Chemical synchronization involves the diffuse release of neurotransmitters like dopamine and acetylcholine, causing transmission delays of several milliseconds. Electromagnetic synchronization encompasses action potentials, electrical gap junctions, and ephaptic coupling. Electrical gap junctions enable rapid synchronization within cortical GABAergic networks, while ephaptic coupling allows structures like axon bundles to synchronize through extracellular electromagnetic fields, surpassing the speed of chemical processes. Quantum synchronization is hypothesized to involve ion coherence during ion channel passage and the entanglement of photons within the myelin sheath. Unlike the finite-time synchronization seen in chemical and electromagnetic processes, quantum entanglement provides instantaneous non-local coherence states. Neurons might have evolved from slower chemical diffusion to rapid temporal synchronization, with ion passage through gap junctions within cortical GABAergic networks potentially facilitating both fast gamma band synchronization and quantum coherence. This mini-review compiles literature on these three synchronization types, offering new insights into the physiological mechanisms that address the binding problem in neuron assemblies.

## Introduction

**Figure 1:**
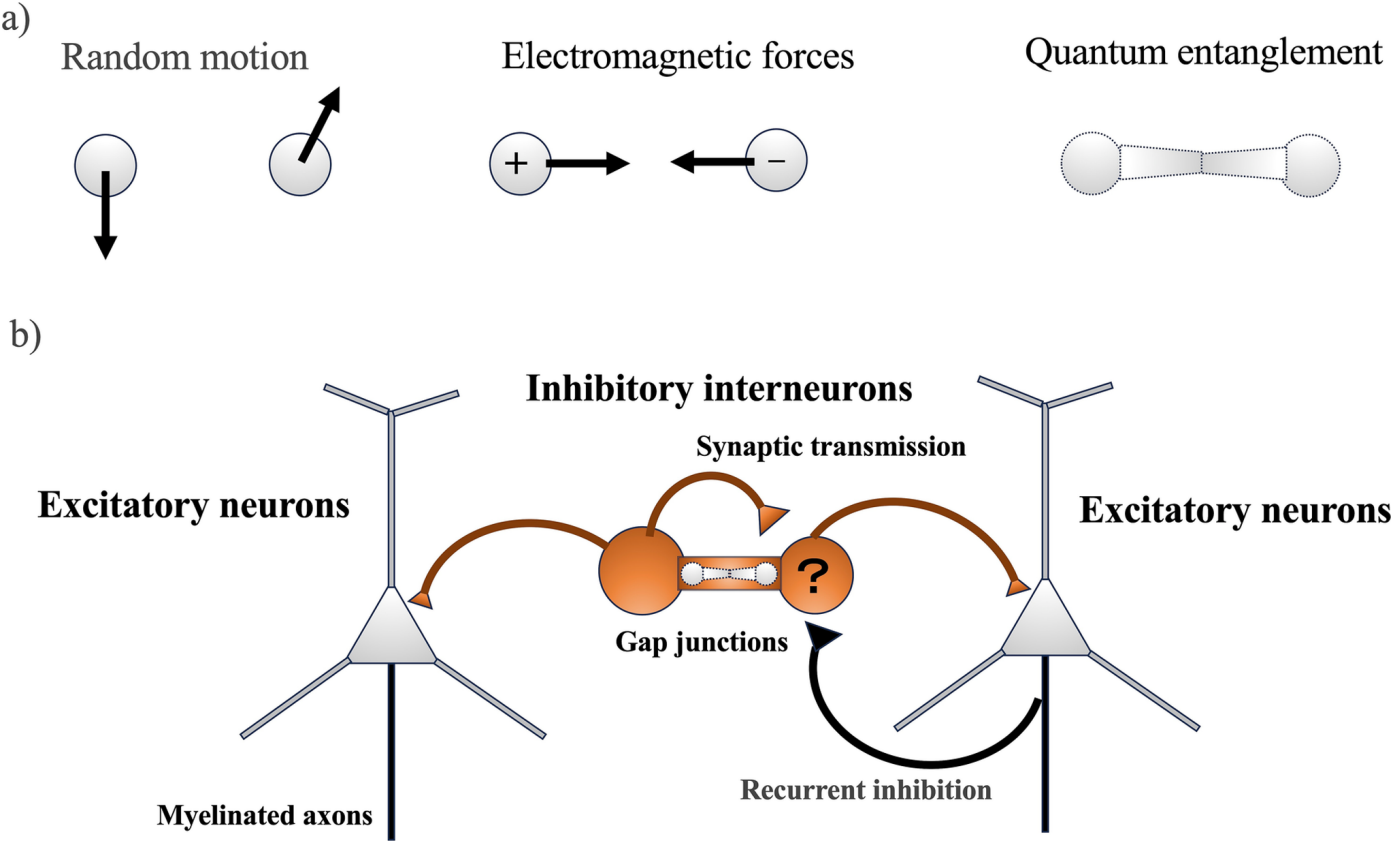
**(A)** The motion of two particles (the minimum number of interactions) influencing molecular evolution in neurons: The movements of the two particles could potentially affect molecular evolution in neurons through three main types of motion: (1) random motion, (2) electromagnetic force, and (3) quantum entanglement. **(B)** Neural circuitry in the cerebral cortex (simple schematic): Inhibitory interneurons function as the origin of rhythm and synchronization, generating regular inhibitory postsynaptic potentials in numerous excitatory neurons. This, combined with feedback inhibition from excitatory neurons, leads to the generation of gamma oscillations, which are intrinsic neural activities characterized by fast rhythms and synchronization. Quantum entanglement might occur in ion channels including gap junctions, where ions moving at high speeds could gather at gap junctions and cause gamma band synchronization. However, it should be noted that ion coherence in gap junctions has not yet been demonstrated.

Kimura’s “Neutral Theory of Molecular Evolution,” proposed in 1968 and further developed in 1983, posits that most DNA (deoxyribonucleic acid) mutations are neutral and spread through populations by chance rather than by natural selection (Kimura, 1968, 1983). This theory is supported by the observation that pseudogenes, which are non-functional segments of DNA, evolve the fastest due to their accumulation of neutral mutations. This aligns with the law of increasing entropy, suggesting that random DNA mutations significantly influence the rate of evolution across different species. While this theory adequately explains the molecular evolution rates of many organisms based on random motion, understanding the evolution of neurons might require considering additional factors such as electromagnetic forces and quantum entanglement (Figure 1A). Neurons might have adapted to environments rich in electromagnetic rhythms and utilize quantum entangled states in specific, noise-free conditions.

Contrary to the rapid evolution of pseudogenes, genes expressed in the brain, especially those in the cortical regions, evolve much more slowly than those in other tissues and subcortical areas (Tuller et al., 2008). This slower rate of evolution indicates functional constraints due to higher-order processes. One of the significant innovations in neuronal evolution is the development of higher brain functions that integrate information into a cohesive system. Similar to essential housekeeping genes that evolve slowly to maintain crucial life functions (Zhang and Li, 2004), genes involved in critical neuronal molecules related to information integration might also evolve slowly. The cortical regions, for instance, are characterized by gamma band oscillations, which are crucial for high-frequency synchronization in neural circuits (Cardin et al., 2009). These oscillations are facilitated by excitatory neurons with recurrent inhibition and inhibitory interneurons connected by gap junctions (Figure 1B) (Fukuda et al., 2006). Dysfunction in these genes might lead to neuropsychiatric disorders such as schizophrenia, which is associated with impaired gamma band synchronization and the dysfunction of cortical GABAergic inhibitory interneurons (Uhlhaas and Singer, 2010; Hirano and Uhlhaas, 2021).

To understand molecular evolution in relation to higher-order functions like information integration in cortical neural circuits, it is essential to address the binding problem. This problem involves the physiological mechanism by which the brain integrates information processed in different locations, such as color, shape, and motion, into a single perceptual experience. Two major hypotheses addressing the binding problem are the synchrony hypothesis [binding by synchrony (BBS)] and the firing rate enhancement hypothesis [binding by firing rate enhancement (BBRE)]. BBS suggests that information is integrated through the synchronous firing of multiple neurons (Singer and Gray, 1995; Singer et al., 1997; Fries, 2015), though this could be challenging when membrane potentials are below the firing threshold or fluctuate spontaneously (Shadlen and Newsome, 1994). BBRE proposes that information is integrated when neurons increase their firing rate simultaneously, forming an assembly and labeling enhanced feature representations with an overall increased firing rate (Roelfsema, 2023). However, BBRE does not fully explain how information within different locations of the assembly is integrated.

To comprehensively understand the molecular evolution related to information integration in neurons, it might be necessary to consider not only random motion but also regular motion induced by electromagnetic forces and state changes due to quantum entanglement (Figure 1A). Furthermore, solving the binding problem requires understanding how neuronal assemblies have evolved the ability to synchronize. This mini-review synthesizes literature on slow chemical synchronization, fast electromagnetic synchronization, and instantaneous quantum synchronization (coherence) in neurons, aiming to explore how these forms of synchronization could contribute to solving the physiological mechanisms of the binding problem by integrating information within neuronal assemblies.

## Slow chemical synchronization

**Figure 2:**
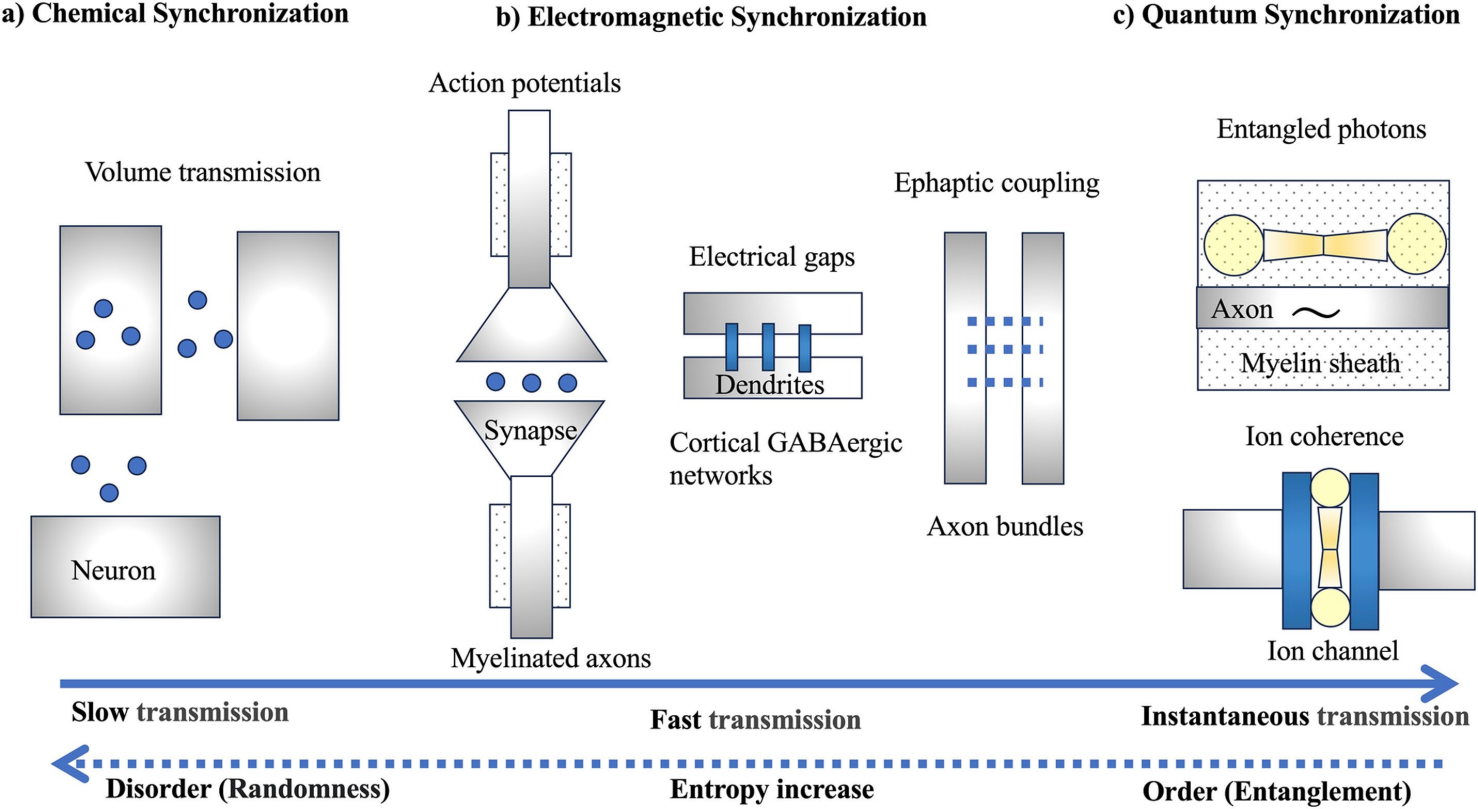
Three types of synchronization: **(A)** chemical synchronization, **(B)** electromagnetic synchronization, and **(C)** quantum synchronization. **(A)** Slow chemical synchronization involves volume transmission, utilizing the random motion of particles. **(B)** Fast electromagnetic synchronization includes not only synaptic transmission through action potentials but also electrical gaps and ephaptic coupling. **(C)** Instantaneous quantum synchronization is proposed to involve entangled photons generated within the myelin sheath and ion coherence generated within ion channels. However, it should be noted that it is not yet known whether ion coherence is generated in electrical gaps, a type of ion channel. The dashed arrows indicate the potential of quantum synchronization, a profound question posed by Schrödinger, to bring order against the increase in entropy in the evolution of life (Schrödinger, 1944).

The emergence of neurons represents a pivotal evolutionary development, but the precise mechanisms of their initial evolution remain uncertain. Approximately 600 million years ago, the ancestors of neurons likely began communicating through the secretion of neuropeptide-containing vesicles, despite lacking the ability to generate electrical activity (Najle et al., 2023). These primitive neuropeptide secretions would have induced contraction responses in nearby cells, allowing rapid reactions to external stimuli. Consequently, early neurons might have primarily utilized chemical synchronization through volume transmission (Figure 2a), disseminating information slowly but simultaneously to multiple neurons (Gianni and Pasqualetti, 2023).

This type of chemical synchronization through volume transmission persists in the mammalian brain. Volume transmission involves the diffusion of neurotransmitters such as acetylcholine, dopamine, norepinephrine, and serotonin from projection neurons in brainstem nuclei, influencing broad brain activities like sleep–wake rhythms, motivation, and emotions (Özçete et al., 2024). Brainstem nuclei such as the ventral tegmental area (VTA) and the pedunculopontine nucleus (PPN) maintain this volume transmission. Unlike the targeted transmission of pyramidal cells in the cerebral cortex, dopaminergic neurons in the VTA release neurotransmitters not only from axon terminals but also from somatodendritic regions, facilitating periodic and diffuse neurotransmitter release (Rice, 2023).

In the VTA, this chemical synchronization is driven by intrinsic rhythmic activity within the 1–5 Hz range, acting as pacemakers for low-frequency local rhythms that synchronize with the prefrontal cortex and hippocampus (Werkman et al., 2001; Bayer et al., 2007; Fujisawa and Buzsáki, 2011; Orzeł-Gryglewska et al., 2015). The rhythmic firing of these neurons induces oscillations in extracellular dopamine concentrations, leading to collective self-inhibition and aiding in neuron synchronization. Thus, the VTA network is viewed as a network of weakly coupled oscillators (van der Velden et al., 2017, 2020). Similarly, the midbrain PPN exhibits spontaneous rhythmic firing at approximately 10 Hz (Takakusaki and Kitai, 1997), which might have contributed to the alpha rhythms observed during dark environment in the cerebral cortex (Shibata et al., 2024), enhancing introspective cognitive abilities characterized by unique alpha rhythms (Supplementary material S1A).

Over evolutionary time, the slow chemical synchronization of peptide-based neurons might have evolved into faster electromagnetic synchronization through synaptic transmission involving action potentials. This evolution improved the precision of information transmission, leading to synaptic wiring characterized by fast and specific one-to-one communication. Modern neural networks incorporate both slow chemical and fast electromagnetic synchronization.

## Fast electromagnetic synchronization

The development of myelin sheaths around axons in complex brains, particularly vertebrates, facilitated rapid nerve impulse conduction, enabling swift responses to environmental stimuli (Castelfranco and Hartline, 2016). High precision in action potential propagation also enhanced memory capabilities through spike-timing-dependent synaptic plasticity (Figure 2b). Even a 1 millisecond delay can shift the phase of gamma oscillations (~100 Hz) by 30°, significantly affecting synaptic plasticity (Pajevic et al., 2014). Thus, the temporal control of information through action potential propagation along axons is a crucial mechanism for synaptic plasticity (Seidl, 2014; Schmidt and Knösche, 2019). Myelination dramatically increased conduction speed (Fields, 2015; Rushton, 1951; Tasaki, 1939), optimizing information transmission efficiency in white matter composed of myelinated axons, influencing the control and synchronization of axonal signal transmission, and affecting alpha wave peak frequencies in the brain (Valdés-Hernández et al., 2010).

Beyond the temporal synchronization of action potentials, electromagnetic synchronization involves networks of cortical GABAergic neurons connected by gap junctions (Figure 2b; Fukuda, 2017). Traditional action potentials in synaptic transmission face delays due to neurotransmitter release and receptor binding, but gap junctions between dendrites of inhibitory interneurons could transmit electrical activity without delay, enhancing synchronization within these networks (Fukuda et al., 2006). Gap junctions are found in the inferior olivary nucleus, vestibular nuclei of rodents, and primate neocortex (Sotelo and Korn, 1978; Sloper and Powell, 1978). These junctions are abundant among dendrites of inhibitory GABAergic interneurons (Kita et al., 1990), enabling high-speed synchronization in the gamma band and contributing to high-frequency electromagnetic synchronization.

Extracellular potentials generated in the cortex, typically around 100 μV, were once considered negligible but have been found to affect adjacent axons as extracellular potentials through ephaptic coupling (Figure 2b), influencing synaptic transmission synchronization (Anastassiou and Koch, 2015). Action potentials propagating along axon bundles could cause synchronization and timing fluctuations of spikes (Ramón and Moore, 1978; Douglas and Blair, 2024), aiding in the integration of subtle odor signals in olfactory neurons (Bokil et al., 2001) and potentially synchronizing distant brain regions (Schmidt et al., 2021). Ephaptic coupling has been proposed to play a role in linking distributed memory networks by forming neural networks (engram complexes) for memory (Pinotsis and Miller, 2023).

The hypothesis of information integration through ephaptic coupling suggests that electric fields could rapidly integrate spatially distributed information, contributing to memory representation and consciousness (Pinotsis and Miller, 2023; Vogeley and Fink, 2003; McFadden, 2020). However, this process remains poorly understood as it involves integrating information within a finite time. Additionally, the intrinsic electric fields of the cortex include synchronization through GABAergic neuron gap junctions, potentially involving ion coherence. Considering quantum entangled states, where information is integrated at the particle level, might help understand information integration across distant brain regions as a natural principle.

Enhancing chemical and electromagnetic synchronization is crucial for brain evolution. In terms of electromagnetic synchronization within neuronal networks, various physiological mechanisms—such as synaptic transmission through action potentials, gap junctions in inhibitory GABAergic neurons, and ephaptic coupling—might play a role in strengthening synchronization. To integrate information across the complex brain system into a unified whole, it is essential to consider not only conventional chemical and electromagnetic synchronization but also quantum synchronization, which facilitates instantaneous information transfer regardless of distance.

## Instantaneous quantum synchronization

Quantum coherence was previously thought to be incompatible with biological systems due to environmental noise, but its potential role in various biological processes is now recognized. Notable examples include electron energy transport in photosynthesis (Engel et al., 2007) and magnetic reception in migratory birds through the radical pair mechanism (Ritz et al., 2000). Despite the growing interest in quantum biology (McFadden and Al-Khalili, 2018), skepticism remains regarding the functional role of transient quantum synchronization in the brain’s complex environment at room temperature. One hypothesis suggests that Posner molecules, composed of calcium and phosphate within neurons, could function as excellent memory bits. These molecules might maintain quantum entanglement for over a day at room temperature, contributing to information transmission and storage (Fisher, 2015; Swift et al., 2018). However, this hypothesis has been experimentally challenged by anesthesia studies (Chen et al., 2020). Similarly, the hypotheses discussed below remain speculative and might be disproven in future studies.

### Ion selectivity and quantum coherence in ion channels

Ion selectivity in ion channels plays a crucial role in the evolution of neurons by enabling precise control over the flow of electrical signals. This selectivity allows specific ions, such as sodium and potassium, to freely pass through the small pores in the cell membrane (Hille, 2022). The physiological mechanisms underlying this selectivity are understood through physical mechanisms, such as the size relationship between the channel pores and the ions, as well as hydration by water molecules (Hille, 1971; Armstrong, 1969). Potassium ion channels are regulated to allow only potassium ions to flow across the membrane, while preventing other ions, particularly sodium ions, from doing so (Doyle et al., 1998). Despite the selective filter region within the channel allowing only potassium ions to pass and excluding sodium ions, the selectivity is remarkably high, allowing up to one hundred million potassium ions per second, while only one sodium ion can pass for every ten thousand potassium ions. The physiological mechanism involves potassium ions shedding surrounding water molecules before lining up along a narrow path in the filter region and then being rehydrated by water molecules after passing through.

However, the temporal movement of ions has not been fully considered, leaving the mechanisms of ion channel selectivity unresolved (Al-Khalili and McFadden, 2014). Therefore, a model has been proposed hypothesizing that quantum coherence might be involved in maintaining the high throughput rate of potassium ions within the highly selective filter of ion channels (Figure 2c; Seifi et al., 2022; Seifi et al., 2024). This model suggests that quantum coherence is necessary to resolve the paradox of achieving both very high throughput rates and extremely high selectivity. Nevertheless, maintaining quantum coherence poses challenges because potassium ions easily lose coherence when interacting with the surrounding environment, such as water molecules, especially at biological temperatures. Additionally, the short time intervals (10–20 nanoseconds) during which ions pass through the channels present difficulties in understanding their role in brain functions such as information integration. Thus, at present, this remains a hypothesis, and further scientific validation is required.

### Entangled photons in the myelin sheath

A recent hypothesis suggests that the vibrations of carbon-hydrogen (C-H) bonds in lipid molecules within the myelin sheath around axons could generate entangled photon pairs, enabling quantum communication (Figure 2c; Liu et al., 2024). While the quantum hypothesis involving the myelin sheath is intriguing, it needs to align with established physiological mechanisms, such as brainwaves (EEG), which have been identified through decades of neuroscience research (Beniczky and Schomer, 2020).

The duration of action potentials along axons is approximately 1 millisecond, which is very short compared to the longer-lasting synaptic potentials (excitatory and inhibitory postsynaptic potentials) that persist after an action potential. These action potentials might be too brief to retain information. In contrast, gamma oscillations generated in the cerebral cortex typically have a frequency of 40 Hz and a duration of 25 milliseconds, allowing them to retain information longer than action potentials. Additionally, EEG signals generated by the synaptic potentials of pyramidal neurons (cortical layers IV-V) also contain gamma band information from cortical GABAergic neurons. Considering these physiological mechanisms suggests that information integration and retention by cortical neural circuits might maintain physiological consistency better than axonal information integration and retention. Therefore, further scientific validation is required to explore the relationship between the quantum hypothesis within the myelin sheath and cortical neural circuits.

## Consistency between electromagnetic and quantum synchronization

Theta-gamma phase-amplitude coupling in EEG is associated with working memory (Papaioannou et al., 2022), while beta-gamma phase-amplitude coupling is related to motor functions (Tanaka et al., 2022). This suggests that in phase-amplitude coupling, the amplitude of high-frequency bands (gamma) contains more information than the phase of low-frequency bands (theta or beta). Additionally, in the cortical visual hierarchy, alpha oscillations propagate in the feedback direction, whereas gamma oscillations influence feedforward processing (Cortes et al., 2020). This indicates that alpha and gamma oscillations play different roles in information processing. Therefore, action potentials along axons alone cannot fully explain the differences between high-frequency and low-frequency bands in solving the binding problem, as action potentials along axons can also contain low-frequency bands.

EEG studies on the phase synchronization index (PSI) have observed temporal correlations (functional connectivity) of EEG signals between distant brain regions. This suggests that EEG synchronization, which idles the entire brain at rest, allows the complex brain to function as a single system (Kawano et al., 2020, 2021). However, since EEG synchronization spans an exponentially changing scale from the neuronal level to the cortical level, it is necessary to examine the relationship between EEG synchronization and quantum synchronization across these scales. The scale levels include: (1) neuronal-level synchronization by 10^0^ to 10^1^ neurons (Gray and Singer, 1989), (2) cortical-level synchronization by 10^1^ to 10^3^ neurons (Roelfsema et al., 1996), (3) multi-regional cortical-level synchronization by 10^4^ to 10^5^ neurons (Bressler et al., 1993), and (4) EEG-level synchronization by 10^6^ to 10^7^ neurons (Rodriguez et al., 1999). Particularly, since EEG synchronization could achieve remote synchronization across the brain through white matter fibers, future scientific validation is needed on the relationship between large-scale brain-wide electromagnetic synchronization and small-scale local Planck-scale quantum synchronization within neurons (Supplementary material S1).

It is well known that general anesthetics cause loss of consciousness by inhibiting synaptic transmission. Recent studies have demonstrated that when anesthetics bind to lipid raft regions within the lipid bilayer, these raft structures expand and disintegrate, leading to the efflux of potassium ions from the cell and the suppression of neuronal excitability (Pavel et al., 2020). Furthermore, anesthetics interfere with high-frequency traveling waves that support consciousness by predominantly promoting low-frequency traveling waves essential for life support (Bhattacharya et al., 2022). Therefore, the photonic coherence hypothesis within the myelin sheath of axon bundles might not sufficiently explain the physiological effects of anesthetics involving the separation of high-frequency and low-frequency brain wave components. On the other hand, considering that electromagnetic synchronization through electrical gaps in cortical neural circuits plays a role in the generation of high-frequency brain waves, it might align with the physiological mechanisms of anesthetics. However, the specific physiological mechanisms by which ion coherence through electrical gaps integrates information held in excitatory neurons into a single piece of information through inhibitory neurons remain poorly understood, presenting numerous challenges.

## Future research

In the evolution of neurons, it might be necessary to investigate the following molecular bases to effectively regulate the synchronization of neuronal assemblies: 1) the probabilistic nature exhibited by neurons and synapses in the brain (volume transmission by neurotransmitters), 2) the strength of synapses that connect neurons (receptor dynamics), 3) the strength of electrical couplings that connect neurons (gap junctions, ephaptic coupling), and 4) the strength of neuronal information coupling (quantum synchronization). Particularly, the integration of information through quantum synchronization (4) still faces many challenges and might require novel approaches to resolve (Supplementary material 2S).

The hypothesis of ion coherence generated by ions passing rapidly through gap junctions is still in its conceptual stage. However, future research might need to comprehensively investigate potential sites of quantum coherence in neural networks, such as Posner molecules within neurons, myelinated axons, and cortical GABAergic network gap junctions. Erwin Schrödinger suggested that life might have evolved by creating order from disorder using negative entropy (negentropy) in defiance of the second law of thermodynamics, which dictates the increase of entropy and the spread of disorder (Schrödinger, 1944). If we consider quantum entanglement as an element of negentropy, quantum synchronization might have been used as negentropy in the evolution of neurons, creating new order over the 3.5 billion years of life’s evolution. This new order in the brain refers to the integration of information that unifies spatially dispersed environmental data temporally. However, this remains an unresolved complex issue. The emergence of ion trap quantum computers has enabled the computation of new “orders” defined by quantum theory using physical models (Figgatt et al., 2019). Similarly, neurons have evolved to increase the frequencies at which they can synchronize firing, allowing for high-frequency oscillations that play crucial roles in sensory and cognitive functions (Noorlag et al., 2022). If ion trap quantum synchronization of ion channels is involved in these high-frequency oscillations, this quantum synchronization might contribute to the integration of sensory and cognitive information. However, since the timescale of quantum synchronization is extremely short (often on the order of picoseconds), it is unclear to what extent it contributes to the integration of sensory and cognitive information. Therefore, future research might elucidate the relationship between high-frequency oscillations and ion trap quantum synchronization.

The concept of spacetime brought about by quantum coherence is not yet fully understood (Hikida et al., 2022). However, if quantum coherence were generating spacetime within neurons, new horizons might open in our understanding of spacetime (Supplementary material S1B). Unlike the integration of information through electromagnetic fields, which requires finite time, the integration of information through quantum bits is instantaneous, regardless of distance. In the future, by unraveling the origins of synchronization in neuron evolution, we might understand the neural circuits necessary for information integration. Ultimately, this could lead to uncovering the profound origins of consciousness that extend beyond the physical space of the brain.

## Conclusion

Neurons might have evolved to enhance adaptability to the environment by integrating information through chemical, electromagnetic, and quantum synchronization. Chemical synchronization, involving the diffusion of neurotransmitters, has a delay of several milliseconds. Throughout evolution, neurons acquired faster methods of synchronization, such as synaptic transmission through action potentials, electrical gap junctions in cortical GABAergic neurons, and ephaptic coupling through extracellular potentials. These advancements allowed for information integration through electromagnetic synchronization, which is faster but still limited in transmission speed. Recently, quantum synchronization involving the instantaneous coherence of neurons has been proposed. This includes ion coherence through selective ion channels and entangled photons in myelinated axons. Quantum entanglement offers the unique characteristic of instantaneous transmission regardless of distance. Understanding the evolutionary origins of neuron synchronization might help elucidate the neural circuit units responsible for integrating information into a unified whole.
