# Supplementary material for: Evolutionary origins of synchronization for integrating information in neurons
Source: Front Cell Neurosci. 2025 Jan 6;18:1525816. doi: 10.3389/fncel.2024.1525816 (PMC11743564; doi:10.3389/fncel.2024.1525816)
Supplement: Supplementary file 1 [file Data_Sheet_1.docx]

Supplementary Material

**Evolutionary Origins of Synchronization for Integrating Information in Neurons**

**Takashi Shibata ^1,2^ *, Noriaki Hattori ^3^, Hisao Nishijo ^4^, Tsutomu Takahashi ^5, 6^, Yuko Higuchi ^5, 6^, Satoshi Kuroda ^1^, Kaoru Takakusaki ^7^**

^１^Department of Neurosurgery, Toyama University Hospital, Japan

^2^ Department of Neurosurgery, Toyama Nishi General Hospital, Japan

^3^ Department of Rehabilitation, Toyama University Hospital, Toyama, Japan

^4^ Faculty of Human Sciences, University of East Asia, Yamaguchi, Japan

^5^ Department of Neuropsychiatry, Graduate School of Medicine and Pharmaceutical Sciences, University of Toyama, Toyama, Japan

^6^ Research Center for Idling Brain Science, University of Toyama, Toyama, Japan.

^7^ The Research Center for Brain Function and Medical Engineering, Asahikawa Medical University, Asahikawa, Japan

*** Correspondence**Corresponding Author: Takashi Shibata, E-mail:sibata@dj8.so-net.ne.jp

**Supplementary information.1**


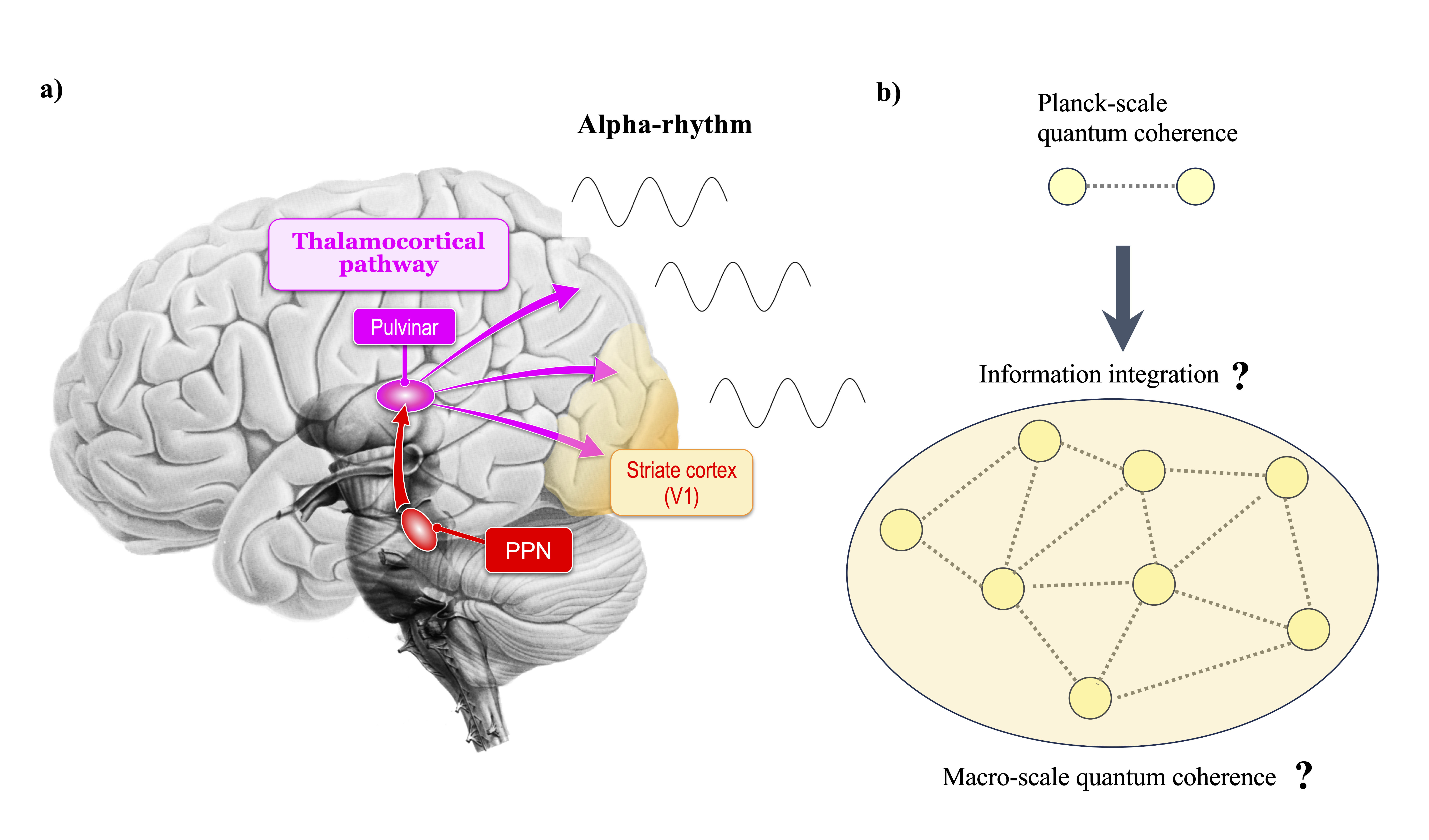


**Consistency between Electromagnetic and Quantum Synchronization**

**a) Schematic of Alpha Rhythms**

The pedunculopontine nucleus (PPN) functions as a pacemaker for the 10Hz rhythm, generating posterior-dominant alpha rhythms through the thalamocortical pathway. In the ancestors of nocturnal mammals, the evolution of alpha rhythms might have significantly enhanced introspective cognitive functions (Shibata et al., 2024). Furthermore, in humans, it has been suggested that the brain network centered on the precuneus developed the ability to perceive the clock function of alpha rhythms marked at 10Hz intervals, enabling the recognition of past, present, and future time (Takahashi and Kitazawa, 2017). However, due to the lack of identified sensory organs (such as eyes or ears) that directly receive the clock function of alpha rhythms, it is possible that neuronal populations evolved to recognize alpha rhythms as a clock through the process of information integration based on high-frequency gamma oscillations in the cerebral cortex. While magnetoreception is known for its ability to sense the geomagnetic field (Shibata et al., 2024), the mechanism by which humans perceive time is not well understood. If humans possess the ability to detect low-frequency electromagnetic oscillations such as alpha rhythms through ion-trap quantum coherence in high-frequency gamma oscillations, it might eventually explain the physiological mechanisms of electromagnetic reception behind human time perception.

**b) Schematic of Quantum Synchronization**

Even if Planck-scale quantum coherence occurs within neurons, it is unclear whether macroscale quantum coherence would emerge throughout the brain to facilitate information integration. Furthermore, the relationship between this macroscale quantum coherence and fundamental electromagnetic rhythms (such as alpha rhythms) is also unknown.

**Supplementary information.2**

Regarding macroscale quantum coherence, Takayanagi, who discovered the "Ryu-Takayanagi formula" for entanglement entropy, suggests the astonishing possibility that in a three-dimensional expanding universe, the macroscopic spacetime might emerge from microscopic quantum bits (the smallest units of spacetime) (Hikida et al., 2022). This remarkable prediction that "the very structure of time in the universe might originate from quantum information" could revolutionize our understanding of spacetime in physics. However, many unknowns and challenges remain, such as the conditions under which spacetime would emerge from quantum entanglement in a four-dimensional expanding universe.

Furthermore, a relationship has been found between electromagnetic synchronization frequencies in the brain and thermal dissipation (entropy production rate) (Sekizawa et al., 2024). High-frequency oscillation modes in the theta and alpha bands, as well as stronger oscillation modes in the delta band, have been observed to significantly contribute to the entropy production rate. However, it is not well understood whether quantum coherence can truly be achieved in special noise-free environments (such as ion channels) within the noisy brain environment characterized by a high entropy production rate. Additionally, it has been suggested that tryptophan in microtubules, which widely permeate the intracellular space as a network, might function as a quantum optical fiber for transmitting quantum signals within the nervous system, even in the noisy brain environment (Babcock et al., 2024). Future research should investigate whether tryptophan can induce photoexcitation absorption from action potentials or electromagnetic waves in the brain and whether the megascale network of tryptophan can function as macroscale quantum coherence over long distances under physiological conditions.

**References**

Babcock, N. S., Montes-Cabrera, G., Oberhofer, K. E., Chergui, M., Celardo, G. L., & Kurian, P. (2024). Ultraviolet Superradiance from Mega-Networks of Tryptophan in Biological Architectures. *The journal of physical chemistry. B*, *128*(17), 4035–4046. <https://doi.org/10.1021/acs.jpcb.3c07936>

Hikida, Y., Nishioka, T., Takayanagi, T., Taki, Y. (2022). Holography in de Sitter Space via Chern-Simons Gauge Theory. Physical review letters, 129(4), 041601. https://doi.org/10.1103/PhysRevLett.129.041601

Sekizawa, D., Ito,S., Oizumi,M. (2024). Decomposing Thermodynamic Dissipation of Linear Langevin Systems via Oscillatory Modes and Its Application to Neural Dynamics. Phys. Rev. X 14, 041003

Shibata, T., Hattori, N., Nishijo, H., Kuroda, S., Takakusaki, K. (2024). Evolutionary origin of alpha rhythms in vertebrates. Frontiers in behavioral neuroscience, 18, 1384340. <https://doi.org/10.3389/fnbeh.2024.1384340>

Shibata, T., Hattori, N., Nishijo, H., Kuroda, S.,  Takakusaki, K. (2024). The origins of light-independent magnetoreception in humans. Frontiers in human neuroscience, 18, 1482872. <https://doi.org/10.3389/fnhum.2024.1482872>

Takahashi, T., Kitazawa, S. (2017). Modulation of Illusory Reversal in Tactile Temporal Order by the Phase of Posterior α Rhythm. The Journal of neuroscience : the official journal of the Society for Neuroscience, 37(21), 5298–5308. https://doi.org/10.1523/JNEUROSCI.2899-15.2017
